# Accounting for Changing Structure in Functional Network Analysis of TBI Patients

**DOI:** 10.3389/fnsys.2020.00042

**Published:** 2020-08-07

**Authors:** John Dell'Italia, Micah A. Johnson, Paul M. Vespa, Martin M. Monti

**Affiliations:** ^1^Department of Psychology, University of California, Los Angeles, Los Angeles, CA, United States; ^2^Brain Injury Research Center (BIRC), Department of Neurosurgery, David Geffen School of Medicine at UCLA, Los Angeles, CA, United States

**Keywords:** network analysis, exponential random graph model, functional magnetic resonance imaging, coma, disorders of consciousness

## Abstract

Over the last 15 years, network analysis approaches based on MR data have allowed a renewed understanding of the relationship between brain function architecture and consciousness. Application of this approach to Disorders of Consciousness (DOC) highlights the relationship between specific aspects of network topology and levels of consciousness. Nonetheless, such applications do not acknowledge that DOC patients present with a dramatic level of heterogeneity in structural connectivity (SC) across groups (e.g., etiology, diagnostic categories) and within individual patients (e.g., over time), which possibly affects the level and quality of functional connectivity (FC) patterns that can be expressed. In addition, it is rarely acknowledged that the most frequently employed outcome metrics in the study of brain connectivity (e.g., degree distribution, inter- or intra-resting state network connectivity, and clustering coefficient) are interrelated and cannot be assumed to be independent of each other. We present empirical data showing that, when the two points above are not taken into consideration with an appropriate analytic model, it can lead to a misinterpretation of the role of each outcome metric in the graph's structure and thus misinterpretation of FC results. We show that failing to account for either SC or the inter-relation between outcome measures can lead to inflated false positives (FP) and/or false negatives (FN) in inter- or intra-resting state network connectivity results (defined, respectively, as a positive or negative result in network connectivity that is present when not accounting for SC and/or outcome measure inter-relation, but becomes not significant when accounting for all variables). Overall, we find that unconscious patients have lower rates of FP and FN for within cortical connectivity, lower rates of FN for cortico-subcortical connectivity, and lower rates of FP for within subcortical connectivity. These lower rates in unconscious patients may reflect differences in their triadic closure and SC metrics, which bias the interpretations of the inter- or intra-resting state network connectivity if the SC metrics and triadic closure are not modeled. We suggest that future studies of functional connectivity in DOC patients (i) incorporate where possible SC metrics and (ii) properly account for the intercorrelated nature of outcome variables.

## 1. Introduction

Over the last 15 years, the study of the functional organization of the human brain under no overt task-set (i.e., so-called resting state fMRI, rsfMRI; Raichle et al., 2001; Van Dijk et al., 2010), has given rise to an explosion in the study of the relationship between the functional brain network properties and cognitive variables (e.g., Zou et al., 2013; Reineberg et al., 2015), biological variables (e.g., Dosenbach et al., 2010; Wang et al., 2013), and disease (e.g., Sorg et al., 2007; Hacker et al., 2012; Pandit et al., 2013; Werner et al., 2014). Similarly, this approach has also been applied toward the understanding the neural underpinnings of consciousness and its disorders (e.g., Monti et al., 2013; Chennu et al., 2014; Crone et al., 2015, 2018; Demertzi et al., 2019).

Despite the popularity and appeal of using network-based description of brain function to assess task-free neuroimaging data, there are a number of important challenges that must be addressed. We have previously made the case for four main shortcomings in current “standard” approach to applying graph-theoretic methods to task-free neuroimaging data, some of which relate to the implementation of the method itself, while others relate to peculiarities of Disorders of Consciousness (DOC) data (cf. Dell'Italia et al., 2018). In this work, we start by providing context on the four shortcomings and then focus on two specific problems which we empirically show lead to inflated rates of false positives and false negatives by virtue of mis-specifying the model.

The first problem we discussed in our prior work (i.e., Dell'Italia et al., 2018) relates to the fact that network properties are typically estimated on the basis of *sparse* matrices—that is to say, on networks where each node only connects to a subset of other nodes, as opposed to fully connected networks where each node is connected to all other nodes. Functional networks, however, conventionally obtained by correlating across time-courses of a large number of regions of interest (ROI), are fully connected. To overcome this issue, it is conventional to make functional networks sparse by selecting a single arbitrary level of sparseness (so-called proportional thresholding), an arbitrary minimum correlation strength *r* below which connections are set to 0 (so-called absolute thresholding), or by computing network summary statistics over several different sparsity levels, typically between the maximum sparsity of 2 × *log*(*N*), with *N* being the number of nodes in the network, which guarantees that networks are estimable, and the lowest level of sparsity that still yields a network with small-world characteristic σ no lower than 1 (Watts and Strogatz, 1998). Importantly, however, summary measures can only be compared across networks that have the same number of connections (i.e., sparsity), thus leading to the requirement to impose the same sparsity across groups (e.g., healthy volunteers vs. severe brain injury patients, minimally conscious state vs. vegetative state patients) or different time-points along the recovery trajectory (e.g., acute vs. chronic), which are very likely to have different levels of “natural density” (cf., Dell'Italia et al., 2018), thus biasing results toward whichever set of networks happens to have natural density closest to the enforced common density level. In response to this issue, some have proposed the use of complex networks (Rubinov and Sporns, 2011; Fornito et al., 2013, 2016, i.e., networks that are fully connected and feature both positive and negative edges). To date, however, this approach has only found very limited application (≈7%; Nielsen et al., 2013).

Second, it is conventional to treat network outcome statistics (e.g., nodal degree, clustering coefficient, etc) as independent of each other. Yet, these outcome statistics are typically interrelated, which can lead to spurious results. To explain, three generative processes are believed to be key in generating a network's topological structure: sociality, selective mixing, and triadic closure. Sociality, a process that operates at the single-node level, refers to the propensity of some nodes to generate more edges than others (e.g., the propensity of different individuals to make friends; Goodreau et al., 2009). Selective mixing, a dyadic-level process, refers to the propensity of an edge to form between two nodes on the basis of some nodal attributes (e.g., the propensity of an individual to make friends with other individuals of the same political or religious persuasion; McPherson et al., 2001; Goodreau et al., 2009). Assortative mixing indicates a greater propensity for edges to form between nodes of the same category whereas dissortative mixing indexes the opposite pattern. Finally, triadic closure, a triadic-level process, refers to the propensity of an edge to form between two nodes *p* and *q* that are each already connected to the same third node *r* (e.g., the propensity of friends of friends to also be friends; Goodreau et al., 2009). While it is often assumed that each outcome metric maps in a one-to-one fashion to the generative processes, this is typically not the case (cf., Figure 2 in Goodreau et al., 2009). Clustering coefficient, for example, defined as the average fraction of a node's neighbors that are also neighbors of each other (Watts and Strogatz, 1998), is often employed as an outcome measure of triadic closure. Indeed, the greater the triadic closure, the greater the clustering coefficient. Yet, selective mixing can also affect the clustering coefficient: when edges are more likely to form between nodes of the same category (i.e., assortative mixing), closure of the triangle is also more likely to occur (leading to a greater clustering coefficient). To exemplify, friends of friends that share membership in a group (e.g., a synagogue, a book club), are more likely to be—themselves—friends, as compared to friends of friends who do not share such a group membership (i.e., dissortative mixing). In the face of this interdependence between outcome metrics, independent testing of the metrics is not advisable (albeit very frequent) since it can lead to misinterpretation and spurious results.

The third major problem in applying network analysis methods to DOC concerns the fact that there are currently no methods for incorporating structural connectivity in the characterization of functional networks. Structural connectivity plays an important role in shaping the functional connectivity that someone can express (cf., Messé et al., 2015,?; Finger et al., 2016). In the context of DOC patients, the high heterogeneity in underlying brain damage both within and across diagnostic categories, as well as the potential for compensatory neuroplastic mechanisms over time (Voss and Schiff, 2009; Demertzi et al., 2011), makes it all the more important that structural information be incorporated in the analysis of functional networks, not to make spurious inferences (see Dell'Italia et al., 2018).

Finally, although we will not discuss the topic in the present work, current analytic frameworks do not allow assessing the dynamics of network change over time. Rather, they rely on the comparison of static characterization of brain networks at different time-points along the recovery trajectory (see Dell'Italia et al., 2018).

In what follows, we adopt the powerful and flexible framework of exponential random graph models (ERGM; Hunter et al., 2008) to estimating network statistics to empirically show that, in patients recovering from coma and a patient population and in healthy volunteers (HCP; Van Essen et al., 2013), problem #2 and problem #3, above, lead to false positives (FPs) and false negatives (FNs) in the estimated networks and that, in the patients, FPs and FNs occur at different rates depending on level of consciousness and time since injury (i.e., acute and 6-month follow-up). We define FPs and FNs by comparing four different models with terms for triadic closure and/or SC included to a base model without those terms. A FP is identified as the base model having a significant parameter estimate (PE) for a specific type of mixing (e.g., within default network connectivity) compared to a model that includes an outcome metric for triadic closure (or including SC metrics) produces a non-significant PE for that specific type of mixing. While a FN is identified as a non-significant PE for a specific type of mixing compared to a model that includes an outcome metric for triadic closure (or including SC metrics) produces a non-significant PE for that specific type of mixing. Thus, we are identifying the effects omitting a triadic closure term (i.e., the GWESP effect), SC metrics (i.e., the structural effect), or both triadic closure and SC metrics (i.e., the interaction effect) on FP and FN rates for selective mixing of resting state connectivity. In our current study, we focused on rate differences of FPs and FNs for selective mixing because much of the DOC research is focused on key regions that form the physical substrate of consciousness. Overall, leaving out important generative processes will produce many FPs and FNs, but rate differences will produced biases in the FPs and FNs that may be interpreted as true differences in patient populations. Thus, we wanted to explore the possibility of rate differences with three questions: (1) What are the rate differences in FPs and FNs for HCP participants during resting state imaging days, (2) what are the rate differences in FPs and FNs for unconscious and conscious patients, and (3) what are the rate differences in FPs and FNs for conscious acute and chronic patients?

## 2. Methods

Our preprocessing remained consistent with our previous study of the single patient (for full details see Dell'Italia et al., 2018). The diffusion data were preprocessed using the following pipeline: DWI preprocessing (brain extraction and bias field correction), registrations (i.e., linear registration to patient's anatomical and non-linear registration to the MNI template using Advanced Normalization Tools; Avants et al., 2008, 2011), probabilistic tractography (i.e., FSL's probtrackx2; Behrens T. et al., 2003; Behrens T. E. et al., 2003) with tractography thresholding (i.e., MANIA; Shadi et al., 2016). The putative preprocessing steps for FC were performed including: brain extraction, slice timing correction, motion correction, band-pass filtering (0.08 ≤ Hz ≤ 0.1), removal of linear and quadratic trends, nuisance regression for signals of non-interest (e.g., motion parameters, white matter, cerebral spinal fluid, and full-brain mean signal), and affine registration of functional data to MNI template (Advanced Normalization Tools; Avants et al., 2008, 2011). The brain network construction remained unchanged using 154 ROIs spanning the cortex, sub-cortical nuclei, cerebellum and brainstem. This parcellation scheme, which was defined independently of our data, is made freely available by Craddock et al. (2012). Additionally, we used the Oxford thalamic connectivity atlas (Behrens T. et al., 2003) to further refine the parcellation of the thalamus from 6 to 14 and we parcellated the basal ganglia into 6 ROIS (caudate, putamen, and globus pallidus each in the left and right hemispheres) for a total of 154 ROI (i.e., 134 Craddock ROIs, 6 basal ganglia ROIs, and 14 Thalamic ROIs). All the HCP data were downloaded with the preprocessing completed using the miniminally processed pipeline (Glasser et al., 2013; Van Essen et al., 2013). Functional connectivity was assessed with a partial correlation method using the Markov Network Toolbox (MoNeT; Narayan et al., 2015) in MATLAB. MoNeT is a tool which combines a penalized maximum likelihood estimation with a resampling-based model selection procedure in order to find the most stable level of sparse brain graph given a set of time-dependent measurements. Each fMRI time series is bootstrapped and resampled in order to estimate the within-subject variablity and a random penalization is applied iteratively to find the most stable solution. This method attempts to reduce the spurious connections that occur from indirect sources, which plague any method using Pearson's R method. Thus, each patient or participant has their own sparse connectivity from direct sources. This estimation procedure was used on both the HCP participants and the patients with DOC. While there are differences in signal quality and exact preprocessing steps between the HCP participants and our patient cohort, our comparisons involved only within HCP participants (across imaging sessions) and within patients (across imaging sessions). Any biases from increased signal quality or differences in preprocessing steps should be controlled for by comparing the data to other data with equal parameters (i.e., within HCP participants and within patients with DOC).

### 2.1. Human Connectome Project Participants

The data for this analysis was taken from the HCP, which is a public repository of high quality structural and functional MR data in a large set of healthy volunteers. For the purposes of this study, we selected a subset of the data (*N* = 12) so to match the characteristics (i.e., age and gender) of the the final set of patients. These HCP participants were randomly sampled from the S1200 (*n* = 9) release, the Q3 release (*n* = 1), S500 (*n* = 1), and S900 (*n* = 1) releases to best match the age and gender of the patients at the time of their injury (see Table 1 for the patients that were matched and see Table 2 for their demographics). These HCP participants only had a single DWI imaging session, which we will use for both days of resting state data.

**Table 1:** Patients' functional MRI parameters.

	**Matched**	**Acute**	**Chronic**	**Acute**	**Chronic**	**Acute**	**Chronic**	**Acute**	**Chronic**
	**MRI**	**TR count**	**TR count**	**slice thickness**	**slice thickness**	**TR**	**TR**	**TE**	**TE**
P054	Yes	200	200	3.4375 × 3.4375 × 3 mm, 40 Slices	3.4375 × 3.4375 × 3 mm, 40 Slices	3,000 ms	3,000 ms	25 ms	25 ms
P055	Yes	200	200	3.5 mm isotropic, 52 Slices	3.4375 × 3.4375 × 3 mm, 64 Slices	3,000 ms	3,000 ms	25 ms	25 ms
P066	No	200	200	3.4375 × 3.4375 × 3 mm, 64 Slices	3.4375 × 3.4375 × 3 mm, 50 Slices	3,000 ms	3,000 ms	25 ms	25 ms
P069	Yes	200	200	3.4375 × 3.4375 × 3 mm, 50 Slices	3.4375 × 3.4375 × 3 mm, 52 Slices	3,000 ms	3,140 ms	25 ms	25 ms
P074	Yes	200	200	3.4375 × 3.4375 × 3 mm, 50 Slices	3.4375 × 3.4375 × 3 mm, 50 Slices	3,000 ms	3,000 ms	25 ms	25 ms
P079	Yes	200	200	3.4375 × 3.4375 × 3 mm, 50 Slices	3.4375 × 3.4375 × 3 mm, 50 Slices	3,000 ms	3,000 ms	25 ms	25 ms
P084	Yes	200	200	3.4375 × 3.4375 × 3 mm, 50 Slices	3.4375 × 3.4375 × 4.25 mm, 37 Slices	3,000 ms	2,006 ms	25 ms	25 ms
P085	Yes	200	200	3.4375 × 3.4375 × 3 mm, 40 Slices	3.4375 × 3.4375 × 4.25 mm, 37 Slices	3,000 ms	2,006 ms	25 ms	25 ms
P089	Yes	200	200	3.00 × 3.00 × 3.99, 33 Slices	3.4375 × 3.4375 × 4.25 mm, 37 Slices	2,000 ms	2,006 ms	30 ms	25 ms
P092	Yes	300	300	3.4375 × 3.4375 × 4.25 mm, 37 Slices	3.4375 × 3.4375x4.25 mm, 37 Slices	2,000 ms	2,006 ms	25 ms	25 ms
P096	Yes	300	300	3.4375 × 3.4375 × 4.25 mm, 37 Slices	3.4375 × 3.4375 × 4.25 mm, 37 Slices	2,000 ms	2,006 ms	25 ms	25 ms
P099	Yes	300	300	3.4375 × 3.4375 × 4.25 mm, 37 Slices	3.4375 × 3.4375 × 4.25 mm, 37 Slices	2,000 ms	2,006 ms	25 ms	25 ms

*The functional MRI parameters are tabulated for each patient. These parameters' descriptions are the same as the DWI paramters's descriptions, except they are for the functional MRI imaging session. An additional parameter for the number to TRs are tabulated under TR count*.

**Table 2:** Patients' demographics.

				**Acute**	**Chronic**	**Delta**	**Acute**	**Acute**	**Chronic**	
	**Gender**	**Cause of injury**	**Age at injury**	**TSI**	**TSI**	**TSI**	**GCS**	**GOS-E**	**GOS-E**	**Group**
P054	Female	Fall	36+	25	186	161	7 (E:2, V:1, M:4)	2	8	Unconscious
P055	Male	Fall	36+	1	195	194	6 (E:1, V:1, M:4)	2	4	Unconscious
P066	Male	Bicycle vs. Automobile	36+	0	222	222	7 (E:2, V:1, M:4)	2	5	Unconscious
P069	Male	Fall	36+	1	186	185	7 (E:2, V:1, M:4)	2	8	Unconscious
P074	Female	Automobile accident	18–25	1	180	179	8 (E:1, V:2, M:5)	3	8	Conscious
P079	Female	Pedestrian vs. Automobile	18–25	2	181	179	7 (E:3, V:3, M:1)	2	7	Unconscious
P083	Male	Pedestrian vs. Automobile	18–25	1	180	179	3 (E:1, V:1, M:1)	2	8	Unconscious
P084	Female	Automobile accident	18–25	5	183	178	3 (E:1, V:1, M:1)	2	3	Unconscious
P085	Female	Fall	36+	2	177	175	15 (E:4, V:5, M:6)	3	7	Conscious
P086	Male	Fall	36+	26	221	195	6 (E:1, V:1, M:4)	3	5	Conscious
P089	Male	Fall	36+	17	181	164	10 (E:2, V:1, M:5)	3	3	Conscious
P092	Male	Automobile accident	18-25	1	158	157	10 (E:4, V:1, M:5)	2	4	Unconscious
P096	Male	Automobile accident	26-30	37	176	139	3 (E:1, V:1, M:1)	3	4	Conscious
P097	Male	Fall	18–25	17	170	153	8 (E:2, V:1, M:5)	3	6	Conscious
P099	Male	Bicycle vs. Automobile	18–25	18	184	166	8 (E:2, V:1, M:5)	3	8	Conscious
P100	Male	Fall	36+	4	173	169	8 (E:2, V:1, M:5)	2	5	Unconscious

*For the 16 patients, the following demographics for each patient is tabulated: gender, cause of injury, age at injury, time since injury for the acute imaging session (Acute TSI), time since injury for the chronic imaging session (Chronic TSI), and the difference in time between the two imaging sessions (Delta TSI). Additionally for each patient, the level of consciousness at the acute imaging session (Acute GCS) and chronic imaging session (Chronic GOS-E) are tabulated. The acute GOS-E is inferred from the GCS scores after the acute imaging session. Finally, these GOS-E scores are used to group patients into two different recovery groups: unconscious and conscious groups*.

#### 2.1.1. HCP Experimental Design

From the HCP dataset, we made use of anatomical (T1-weighted), diffusion (Diffusion Tensor Imaging; DTI), and functional (T2*-weighted) data. T1-weighted images were acquired with a 3D MPRAGE sequence (repetition time [TR] = 2,400 ms, echo time [TE] = 2.14 ms, flip angle [FA] = 8 deg). DTI images were acquired with a spin-echo echo planar sequence (TR = 5,520 ms, TE = 89.5 ms, FA = 78 deg/160 deg, 96 directions). Finally, blood oxygenation level dependent (BOLD) functional image were acquired with a gradient-echo echo planar image (TR = 720 ms; TE = 33.1 ms; FA = 52 deg).

#### 2.1.2. Functional Connectivity Patient Cohort

Of the original 16 patients, 4 patients were excluded due to BOLD artifacts (patient P083), preprocessing errors (patient P086 and P097), or registration errors (patient P100). After these final exclusions there were 12 patients (see Table 1), which seven of these patients were male and five were female. All of these patients were presented with a post-resuscitation GCS during the acute stage of TBI which was transformed into an inferred GOS-E (Crone et al., 2018). Additionally, the GOS-E was assessed at the chronic stage of TBI. Together, the inferred GOS-E and chronic GOS-E were used to split 7 patients in the unconscious group and 17 patients in conscious group (i.e., 5 acute patients and 12 chronic patients).

#### 2.1.3. Patients' Experimental Design

The 16 patients underwent two imaging sessions over the span of at least 158 days to at most 222 days. The first session occurred at most 37 days post injury (see Table 2), and the follow-up session took place 238 days post-injury. At each session the patient underwent (among other clinical and research sequences) anatomical (T1-weighted) and functional (T2^*^-weighted) data protocols. T1-weighted images were acquired with a 3D MPRAGE sequence (repetition time [TR] = 1900 ms, echo time [TE] = 3.43, 1 × 1 × 1 mm). BOLD functional data were acquired with a gradient-echo echo planar image (see Table 1 for the slice thickness, TR count, TE, and TR). Diffusion weighted data were acquired with an echo planar sequence (for number of gradient directions, TR, TE, and slice thickness see Table 3) using a *b*-value of 1,000 and acquiring an additional B0 image. Acute data were acquired on the in-patient 3 Tesla Siemens TimTrio system at the Ronald Reagan University Medical Center for patients P003, P005, P007, P014, P018, P021, P023, P024, P026, P027, P029, P039, and P066, and rest of the patients' acute data were acquired on a 3 Tesla Siemens Prisma system. All the chronic data were acquired on the out-patient 3 Tesla Siemens Prisma system also at the Ronald Reagan Medical Center at the University of California Los Angeles. The study was approved by the UCLA institutional review board (IRB). Informed consent was obtained from the legal surrogate, as per state regulations.

### 2.2. ERGM and Graph Statistics

The core idea underlying ERGM is the estimation of possible network statistics that generate a family of graphs. Edges are treated as a random variable generated by a stochastic process that could have been sampled for a number of possible graphs, which are produced from similar generative processes. A logistic regression with multiple predictor variables is used to estimate the unique contribution of each network statistic defined by:

(1) $$\begin{array}{l} P_{\theta }\left(Y=y\right)=\frac{\exp\left(\theta^{T}g\left(y\right)\right)}{c\left(\theta \right)} \end{array}$$

where θ is a parameter vector that is modeled by g(y) (i.e., vector of graph statistics used in each model). The parameter *c*(θ) is a normalizing constant representing the parameter estimate for all possible graphs (Hunter et al., 2008). This normalizing constant is not able to be analytically solved in most models, instead a Markov Chain Monte Carlo (MCMC) methods is used to sample and estimate the population mean. These methods assume Markovian principles of independent draws and the ability to reach equilibrium. Equilibrium is the state in which any edge that is toggled on or off results in an equally probable graph.

For both the HCP participants' and patients' datasets, we ran 4 ERGM: complete model (i.e., edges term, nodemix for resting state connectivity, the SC metrics, and triadic closure terms), structural model (i.e., edges term, nodemix for resting state connectivity, and the SC metrics), geometrically weighted edged shared partners (GWESP) model (i.e., all terms except SC metrics), and base model (i.e., only the edges term and nodemix for resting state connectivity). The base model was specified as follows, where *P*_θ_(*Y* = *y*):

(2) $$=\frac{\exp(\theta_{1}\text{edges}+\theta_{2}\text{nodemix}(\text{rest})}{c(\theta )}$$

The edges term is used to control for the overall density of each graph allowing for each graph to have a different density. The nodemix(rest) term (i.e., the selective mixing for resting state networks and subcortical regions) creates multiple terms for each possible inter- (e.g., inter-frontoparietal and default network connectivity), intra-resting state network connectivity (e.g., within default network connectivity), inter-subcortical (e.g., thalamo-basal ganlia connectivity), intra-subcortical (e.g., within basal ganglia connectivity), and between subcortical and resting state connectivity (e.g., thalamo-frontroparietal network connectivity.

The structural model includes structural connectivity covariates estimated from the structural connectivity adjacency matrix, where *P*_θ_(*Y* = *y*):

(3) $$=\frac{\begin{array}{l} \exp(\theta_{1}\text{edges}+\theta_{2}\text{nodecov}(\text{degree})+\theta_{3}\text{nodecov}(\text{efficency})\\ \;+\theta_{4}\text{nodematch}(\text{latent})+\theta_{5}\text{nodecov}(\text{cluster})+\theta_{6}\text{nodemix}(\text{rest}) \end{array}}{c(\theta )}$$

The structural model has the same terms (i.e., edges and nodemix) as the base model with three additional nodal covariates (i.e., degree, local efficiency, and nodal clustering coefficient). These nodal covariates incorporate structural connectivity differences into the functional connectivity modeling.

The GWESP model has the same terms (i.e., edges and nodemix) as the base model with a term for triadic closure, where *P*_θ_(*Y* = *y*):

(4) $$\begin{array}{l} =\frac{\exp\left(\theta_{1}\text{edges}+\theta_{2}\text{nodemix}\left(\text{rest}\right)+\theta_{3}\text{gwesp}\left(\text{alpha }=\lambda \right)\right)}{c\left(\theta \right)} \end{array}$$

The GWESP term was added to the edges and nodemix(rest) terms to account for the triadic closure. This term models the number of edge shared partners in each graph, but it applies a geometrically weighted distribution to penalize higher counts of edge shared partners (see Hunter, 2007, for a complete description)

The complete model contains both the structural terms and the GWESP term, where *P*_θ_(*Y* = *y*):

(5) $$=\frac{\begin{array}{l} \exp(\theta_{1}\text{edges}+\theta_{2}\text{nodecov}(\text{degree})+\theta_{3}\text{nodecov}(\text{efficency})\\ \;+\theta_{4}\text{nodematch}(\text{latent})+\theta_{5}\text{nodecov}(\text{cluster})+\theta_{6}\text{nodemix}(\text{rest})+\theta_{7}\text{gwesp}(\text{alpha}=\lambda )) \end{array}}{c(\theta )}$$

These four models will be used to estimate rate differences using a multinomial regression (see section 2.3). Each model was assessed for its overall fit to the observed data. Due to the large number of total ERGM conducted (192 in total across patients and HCP participants), we will only compare for two patients (i.e., P092 in acute stage TBI and P085 in chronic stage TBI) and HCP patients (i.e., HCP002 from the first and HCP008 resting state imaging sessions) for the complete and GWESP models assessed by using goodness of fit (GOF) plots (Hunter et al., 2008). After the model was estimated, a thousand simulations were run from the model statistics that generated 1,000 separate graphs with the generative processes captured from each of the four models. These simulations provided a distribution of mixing terms (see Table 4 for patients, and for HCP participants, see Table 5) and edge shared partners (see Table 6 for patients) that were compared to the original graph's edge probabilities. The mean and variance of these distribution were used to test for differences between the simulated distributions and the original data's distribution. This is to ensure that the model represents a graph similar to the original data that it was modeled from. We will assess the overall model statistics from equation 5 and edge shared partner distributions.

**Table 3:** Patients' DWI parameters.

		**Acute**	**Chronic**	**Acute**	**Chronic**	**Acute**	**Chronic**	**Acute**	**Chronic**
	**Matched MRI**	**Bvec**	**Bvec**	**slice thickness**	**slice thickness**	**TR**	**TR**	**TE**	**TE**
P054	Yes	64	63	2 mm isotropic, 69 Slices	2.125 × 2.125 × 2 mm, 69 Slices	9,000 ms	9,000 ms	90 ms	90 ms
P055	Yes	56	56	2 × 2 × 3 mm, 50 Slices	2.125 × 2.125 × 2 mm, 81 Slices	8,000 ms	9,000 ms	96 ms	90 ms
P066	No	41	38	2 mm isotropic, 81 Slices	2 mm isotropic, 69 Slices	9,300 ms	9,000 ms	90 ms	90 ms
P069	Yes	59	61	2 mm isotropic, 69 Slices	2.125 × 2.125 × 2 mm, 77 Slices	9,000 ms	9,900 ms	90 ms	90 ms
P074	Yes	58	62	2 mm isotropic, 69 Slices	2.125 × 2.125 × 2 mm, 69 Slices	9,000 ms	9,000 ms	90 ms	90 ms
P079	Yes	64	62	2 mm isotropic, 69 Slices	2.125 × 2.125 × 2 mm, 69 Slices	9,000 ms	9,000 ms	93 ms	90 ms
P084	Yes	64	62	2 mm isotropic, 69 Slices	2 × 2 × 3 mm, 52 Slices	9,000 ms	9,000 ms	90 ms	90 ms
P085	Yes	54	62	2 mm isotropic, 69 Slices	2 × 2 × 3 mm, 52 Slices	10,100 ms	9,000 ms	90 ms	90 ms
P089	Yes	60	62	2 × 2 × 3 mm, 50 Slices	2 mm isotropic, 72 Slices	9,500 ms	9,500 ms	90 ms	90 ms
P092	Yes	61	63	2 mm isotropic, 78 Slices	2 mm isotropic, 75 Slices	10,100 ms	9,500 ms	90 ms	90 ms
P096	Yes	45	60	2 mm isotropic, 78 Slices	2 mm isotropic, 78 Slices	10,100 ms	10,100 ms	90 ms	90 ms
P099	Yes	62	64	2 mm isotropic, 78 Slices	2 mm isotropic, 78 Slices	10,100 ms	10,100 ms	90 ms	90 ms

*The following parameters for each DWI imaging session (i.e., acute and chronic) varied from patient to patient due to clinical requirements: the number of gradient directions (Bvec), the slice thickness, the repetition time (TR), and the echo times (TE). Additionally, the matched MRI indicates which patients had the same MRI system in both the acute and chronic imaging sessions*.

**Table 4:** Goodness of fit differences for graph statistics.

	**Complete model**					**GWESP model**			
	**Observed**	**min**	**M**	**max**	***p*-value**	**min**	**M**	**max**	***p*-value**
*HCP001 Rest1*									
Inter-frontoparietal subcortical	11	5	10	19	1	3	8.600	14	0.200
Inter-default visual	82	65	78.700	90	0.800	65	77.400	88	0.200
Inter-default limbic	69	57	64.800	70	0.200	56	68	80	1
Inter-subcortical thalamus	14	13	16.500	20	0.200	3	13	21	1
Inter-default ventral attention	79	75	86.100	100	0.200	62	78.300	86	1
*HCP002 Rest1*									
Edges	2, 044	2, 023	2, 059.600	2, 113	1	1, 901	1, 983.800	2, 064	0.200
Inter-limbic thalamus	20	15	20.500	26	1	9	14.900	22	0.200
Within subcortical	159	144	161.800	173	0.600	112	143.400	169	0.200
Gwesp Fixed, λ=0.6	3, 634.600	3, 593.262	3, 660.370	3, 756.905	1	3, 367.806	3, 521.756	3, 670.710	0.200
*HCP003 Rest1*									
Inter-dorsal attention dorsa attention	7	6	8.500	12	0.200	3	6.700	11	1
Inter-subcortical visual	10	6	8.300	14	0.200	4	10.300	18	0.800
*HCP004 Rest1*									
Within thalamus	23	16	25.900	33	0.400	23	32.200	39	0.200
Within subcortical	138	118	144.300	160	0.400	140	160.600	169	0
*HCP005 Rest2*									
Inter-frontoparietal somatomotor	45	29	38.800	45	0.200	30	43.300	50	1
Within subcortical	174	156	184.100	200	0.200	148	167.400	176	0.600
*HCP006 Rest2*									
Inter-frontoparietal somatomotor	45	29	38.800	45	0.200	30	43.300	50	1
Within subcortical	174	156	184.100	200	0.200	148	167.400	176	0.600
*HCP008 Rest2*									
Inter-limbic thalamus	26	19	23	30	0.400	16	20.800	29	0.200
Inter-somatomotor thalamus	75	64	73.800	87	0.800	60	67.900	76	0.200
Inter-limbic visual	28	15	27.100	37	0.800	18	21.900	27	0
*HCP009 Rest1*									
Inter-limbic subcortical	16	14	17.400	21	1	17	19.200	24	0
Inter-frontoparietal ventral attention	46	35	46.700	52	0.800	45	49.900	57	0.200
Inter-dorsal attention visual	28	21	28.200	38	1	20	24.200	34	0.200
*HCP010 Rest1*									
Inter-basal ganglia default	33	24	31.700	44	0.400	25	29.200	32	0
Inter-default dorsal attention	41	29	40.100	53	0.800	36	44.400	47	0.200
*HCP010 Rest2*									
Inter-basal ganglia limbic	13	7	11.100	18	0.200	8	12.900	21	1
*HCP012 Rest2*									
Inter-basal ganglia ventral attention	18	17	20.800	30	0.200	12	16.200	24	0.600

*The observed column is the original data's values for each graph statistic, while the minimum, mean, maximum, and p-value for the simulated graphs based on each of the ERGM models are displayed. They are the biggest difference between the complete model and the GWESP model. Overall, all the patients' ERGM for the complete model and GWESP effect fit the data well based on the graph statistics modeled but the GWESP model had 4 p < 0.05*.

**Table 5:** Goodness of fit differences for graph statistics.

	**Complete model**					**GWESP model**			
	**Observed**	**min**	***M***	**max**	***p*-value**	**min**	***M***	**max**	***p*-value**
*P054 Acute*									
Edges	1, 796	1, 702	1, 780.300	1, 839	0.800	1, 793	1, 839.300	1, 886	0.200
Intra-limbic	19	15	21.300	28	0.400	18	22.100	28	0.200
Inter-somatomotor-ventral attention	56	50	57.700	69	1	54	63.300	74	0.200
GWESP (fixed, λ = 0.45)	2, 766.980	2, 614.125	2, 741.347	2, 834.899	0.800	2, 761.997	2, 835.273	2, 908.383	0.200
*P084 Acute*									
Inter-frontoparietal-somatomotor	40	28	39	52	1	6	10.400	16	0.200
Inter-frontoparietal-thalamus	16	13	18.250	28	0.700	13	18.200	22	0.200
*P084 Chronic*									
Inter-dorsal attention-limbic	9	9	11.300	14	0.200	2	8	12	1
Within subcortical	153	151	159	172	0.200	127	144.300	161	0.600
*P085 Acute*									
Inter-dorsal attention-ventral attention	12	6	9.700	15	0.200	10	15	21	0.800

*The observed column is the original data's values for each graph statistic, while the minimum, mean, maximum, and p-value for the simulated graphs based on each of the ERGM models are displayed. None of these are bad fits, but they are the biggest difference between the complete model and the GWESP model. Overall, all the patients' ERGM for the complete model and GWESP effect fit the data well based on the graph statistics modeled because neither model produced any p < 0.05 for any graph statistic*.

**Table 6:** Goodness of fit differences for edge shared partners.

	**Complete model**					**GWESP model**				**Complete model**					**GWESP model**			
	**P054 acute**					**P054 acute**				**P084 chronic**					**P084 chronic**			
	**Observed**	**min**	***M***	**max**	***p*-value**	**min**	***M***	**max**	***p*-value**	**Observed**	**min**	***M***	**max**	***p*-value**	**min**	**M**	**max**	***p*-value**
esp0	4	0	0.100	1	0	0	0	0	0	16	0	0.700	2	0	0	1.600	4	0
esp1	20	1	3.200	6	0	1	3.200	6	0	63	42	56.800	73	0.800	50	58.500	67	0.400
esp2	58	50	65	83	0.600	54	74	89	0.400	140	198	231.700	254	0	182	226.200	258	0
esp3	140	226	245.900	269	0	224	252.800	292	0	243	311	341.300	371	0	326	347.500	391	0
esp4	213	357	385	410	0	326	359.100	383	0	275	311	335.200	365	0	316	332.900	353	0
esp5	255	362	384	406	0	360	380.300	401	0	260	200	237.600	277	0.400	217	240.600	267	0.200
esp6	275	276	311.200	351	0	261	292.500	326	0.600	201	113	142.900	167	0	124	149.600	174	0
esp7	245	176	205	231	0	170	198.300	231	0	143	51	76.700	95	0	67	80.100	94	0
esp8	197	89	116.100	147	0	81	110.600	135	0	72	26	35.600	47	0	20	35.700	46	0
esp9	158	54	64.200	78	0	40	60.900	79	0	40	7	14.600	26	0	11	14.700	25	0
esp10	93	26	34.800	45	0	17	28.500	41	0	17	3	7	15	0	3	5.400	9	0
esp11	68	6	14.600	22	0	9	12.900	22	0	12	0	2.500	6	0	0	1.800	3	0
esp12	28	4	6	9	0	1	4.300	7	0	6	0	1	3	0	0	1.100	4	0
esp13	24	1	2.700	5	0	0	2.200	4	0	1	0	0.500	1	1	0	0.100	1	0.200
esp14	11	0	0.900	3	0	0	0.600	3	0	0	0	0	0	1	0	0	0	1

*The observed column is the original data's values for each edge shared partner type, while the minimum, mean, maximum, and p-value for the simulated graphs based on each of the ERGM models are displayed. The edge shared partner types are based on the number of triangles sharing a common edge (e.g., the esp10 term has 10 triangles all sharing common edge). Overall, all the patients' ERGM for the complete model and GWESP effect did not fit the data well based on the graph statistics modeled because both models had at least than 11 of the 14 p < 0.05 for types of edge shared partner type*.

### 2.3. Multinomial Regressions

We compared these 4 ERGMS in three combinations: base model to structural model (i.e., structural effect), base model to GWESP model (i.e,. GWESP effect), and base model to complete model (i.e, interaction effect). The first comparison was to isolate the effects of leaving out structural terms discussed in problem #3. The second comparison was to isolate the effects of leaving out a term that accounts for triadic closure (i.e., the GWESP term) discussed in problem #2. Finally, the third comparison demonstrates the effects of leaving out the structural terms while still accounting for triadic closure (i.e., a combination of problem #2 and #3). We labeled one model as the full model and one as the partial model in each comparison.

To compare the affects of not accounting for specific terms, we tallied the change in PEs when the terms were omitted. If a PE was significant in the full model (i.e., the model with more terms for that specific comparison), but not the partial model (i.e., the base model), we label this as a FN. FP was a PE that was significant in partial model, but not the full model. The true positives (TPs) and true negatives (TNs) are the terms that are significant or non-significant–respectively, in both models. We group the PEs based on whether they belonged to the cortical regions or subcortical to see if within cortical connectivity was affected, within subcortical connectivity, or between cortical to subcortical connectivity.

These tallies of FP and FN were compared for differences between patients grouped based on their level of consciousness at each imaging session using a behavior assessment. During the acute session, patients were evaluated with a post-resuscitation Glasgow Coma Scale (GCS; Teasdale and Jennett, 1974). The GCS has three subscales: eyes opening (E), verbal response (V), and motor response (M). Crone et al. (2018) used the GCS subscales of V and M to transform the GCS scores into the Glasgow Outcome Scale-Extended (GOS-E; Wilson et al., 1998). A patient with a GCS V subscale of less than or equal 3 and a GCS M subscale of less than or equal to 4 were assigned an inferred GOS-E of 2, while a patient with higher scores for GCS V and M were assigned an inferred GOS-E of 3. While DoC diagnoses are typically not made at such an acute stage, a patient with a GOS-E of 2 is consistent with a VS, and patient with a GOS-E of 3 has recovered from VS. This allows organizing patients into two groups (see Table 2): unconscious patients vs. conscious patients. To mirror this in the HCP datasets, we compared the first resting state imaging to the second resting state imaging session.

Using the nnet package Venables and Ripley (2013) in R, we used the mlogit function to multinomial regressions to predict the differences between unconscious patients' FP and FN rates for the cortical groupings (i.e., within cortical connectivity, within subcortical connectivity, and cortico-subcortical connectivity). These cortical groupings are for the mixing of the resting state networks and subcortical structures. The no error for all grouping was the reference group for the outcome variable and the acute conscious patients were the reference group for the predictor variable. For the cortical group there were 6 possible categories predicted, which were FN and FP for each grouping. The same comparisons were conducted for the HCP participants, but there was only one possible comparison between the first and second resting state scan. Finally, we transformed all the logits into odds ratios for reporting and interpretations.

## 3. Results

**Figure 1:**
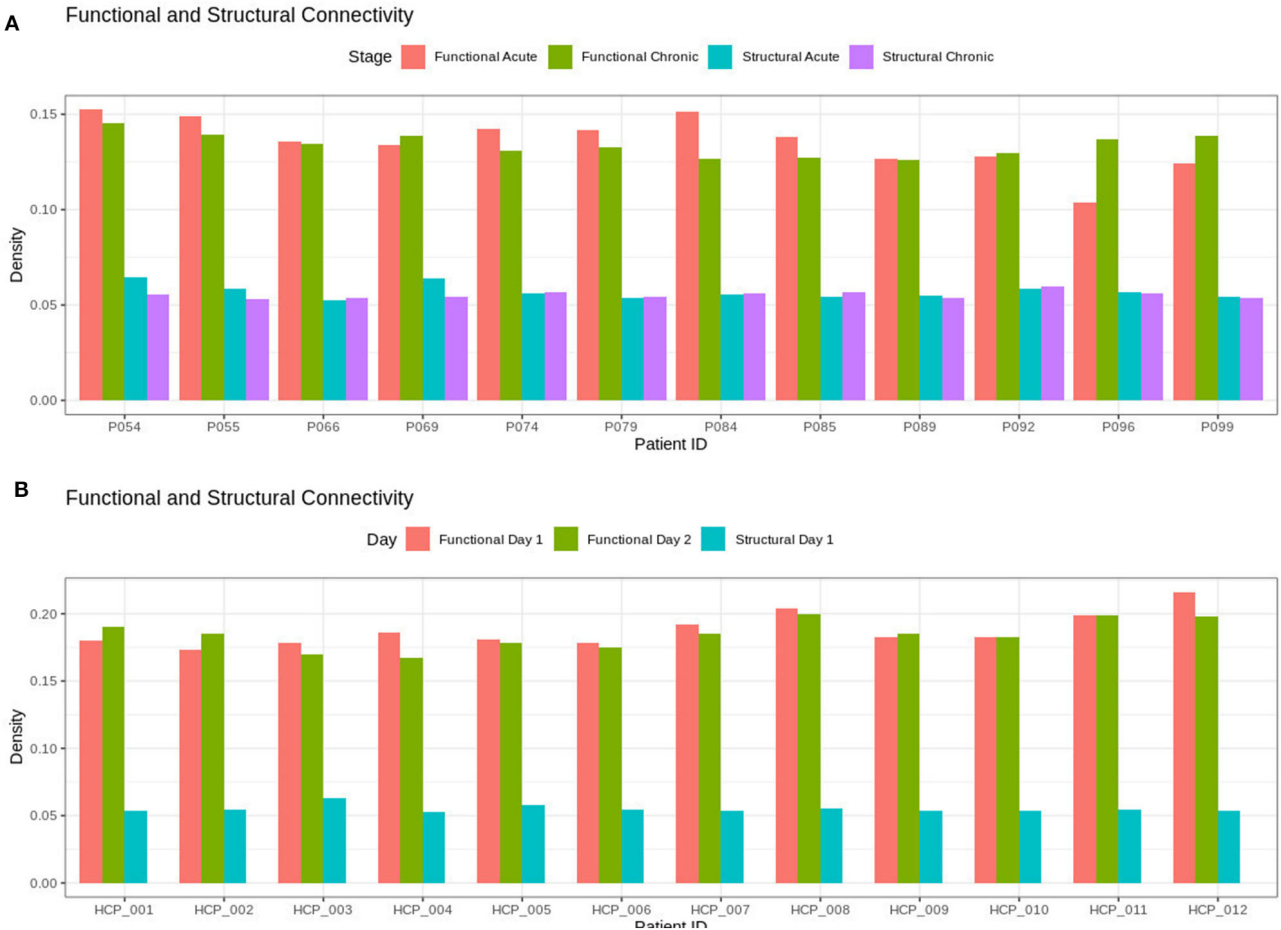
Densities for the functional and structural connectivity. The functional connectivity and structural connectivity was allowed to naturally vary based on the thresholding procedure. In **(A)**, there is no clear difference within the patients when comparing acute and chronic stage of TBI for neither the structural or functional density. In **(B)**, the HCP participants also show no clear difference between resting state imaging on day 1 compared to day 2.

As shown in Figure 1, the brain network construction using MoNeT resulted in different estimated densities. Overall, the density varied between resting state session 1 and session 2 in all participants (except HCP011) within the range between 0.003 to 0.0175. Across HCP participants, the densities ranged from 0.1676 to 0.2159 and the structural connectivity had less variability in the densities of the graphs across subjects ranging from 0.0531 to 0.0632. For the patients, the density of the functional connectivity differed between resting acute session and chronic session in all patients within the range between 0.0019 to 0.0331. Across patients, the densities ranged from 0.1039 to 0.1524 and the structural connectivity had less variability across acute and chronic imaging sessions in the densities (i.e., a difference between 0.005 to 0.0095).

**Figure 2:**
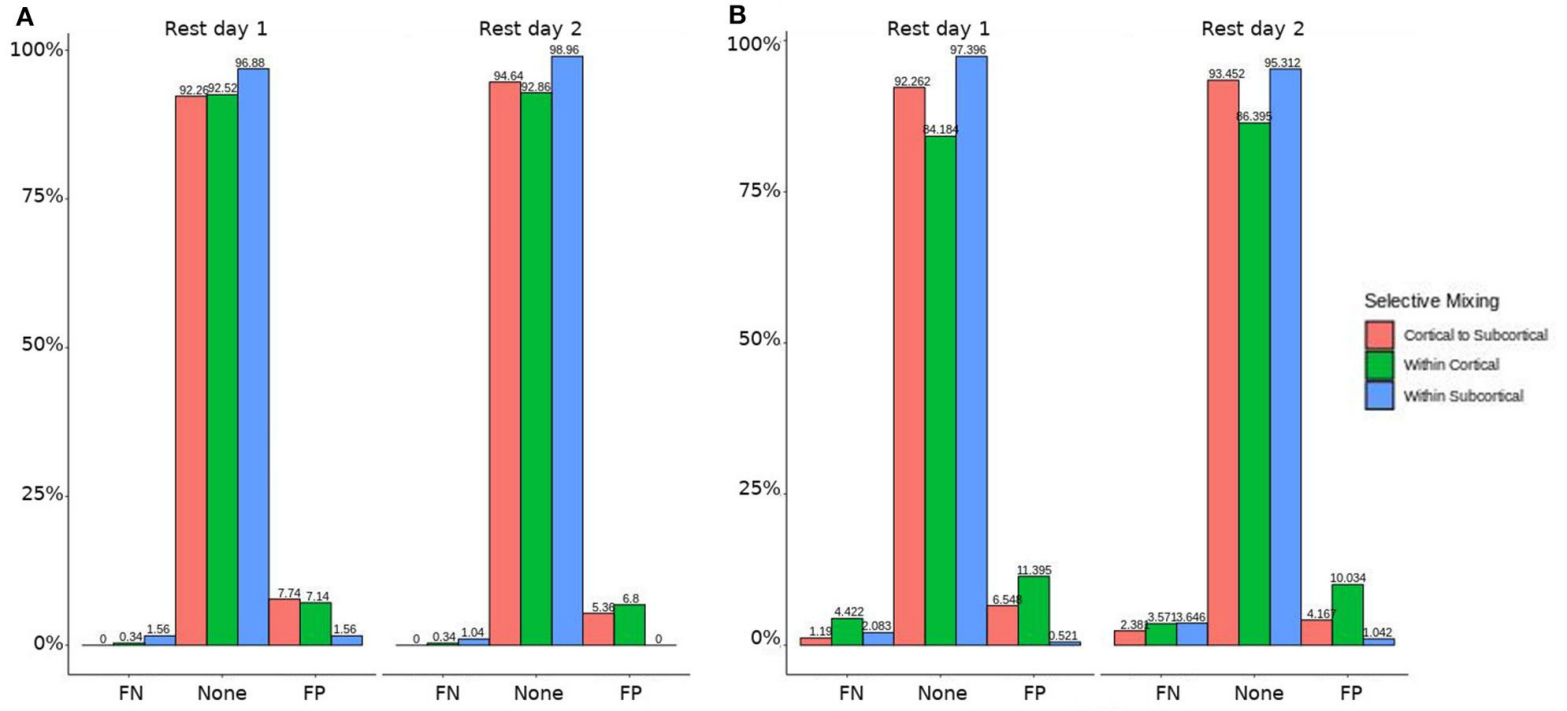
HCP participants model comparisons for the structural and GWESP effects. For the model comparison of base model (i.e., selective mixing only) to GWESP model (i.e., selective mixing with modeling triadic closure), this figure shows, in column **(A)** the FN and FP rates of resting state network nodes grouped by cortical and subcortical regions for each resting state imaging session separated by 1 day. The none category contains both the TP and TN across models. In column **(B)** the effect on the FN and FP rates for leaving out the structural connectivity terms (i.e., structural effect) for the same types of connectivity. These model comparisons revealed no structural or triadic closure effects on the rates of FPs and FNs for rate differences in cortical or subcortical regions' connectivity patterns across the different resting states on 2 separate days.

**Figure 3:**
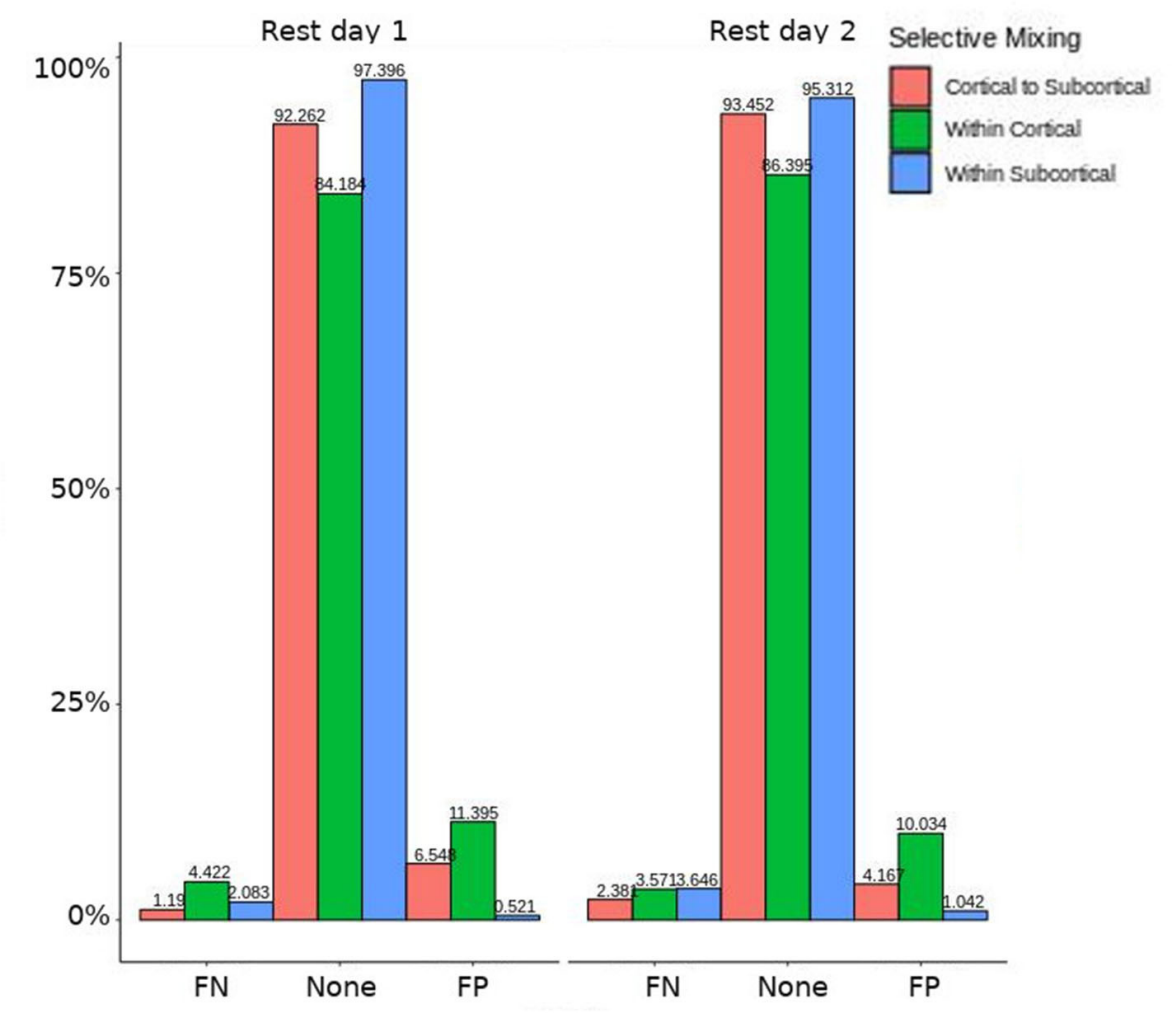
HCP participants model comparison for the interaction effect. For the model comparison of base model (i.e., selective mixing only) to complete model (i.e., selective mixing with modeling both triadic closure and SC), this figure shows the FN and FP rates of resting state network nodes grouped by cortical and subcortical regions for each resting state imaging session separated by 1 day. The none category contains both the TP and TN across models. This comparison revealed no rate differences in cortical or subcortical regions' connectivity patterns across the different resting states on 2 separate days.

**Figure 4:**
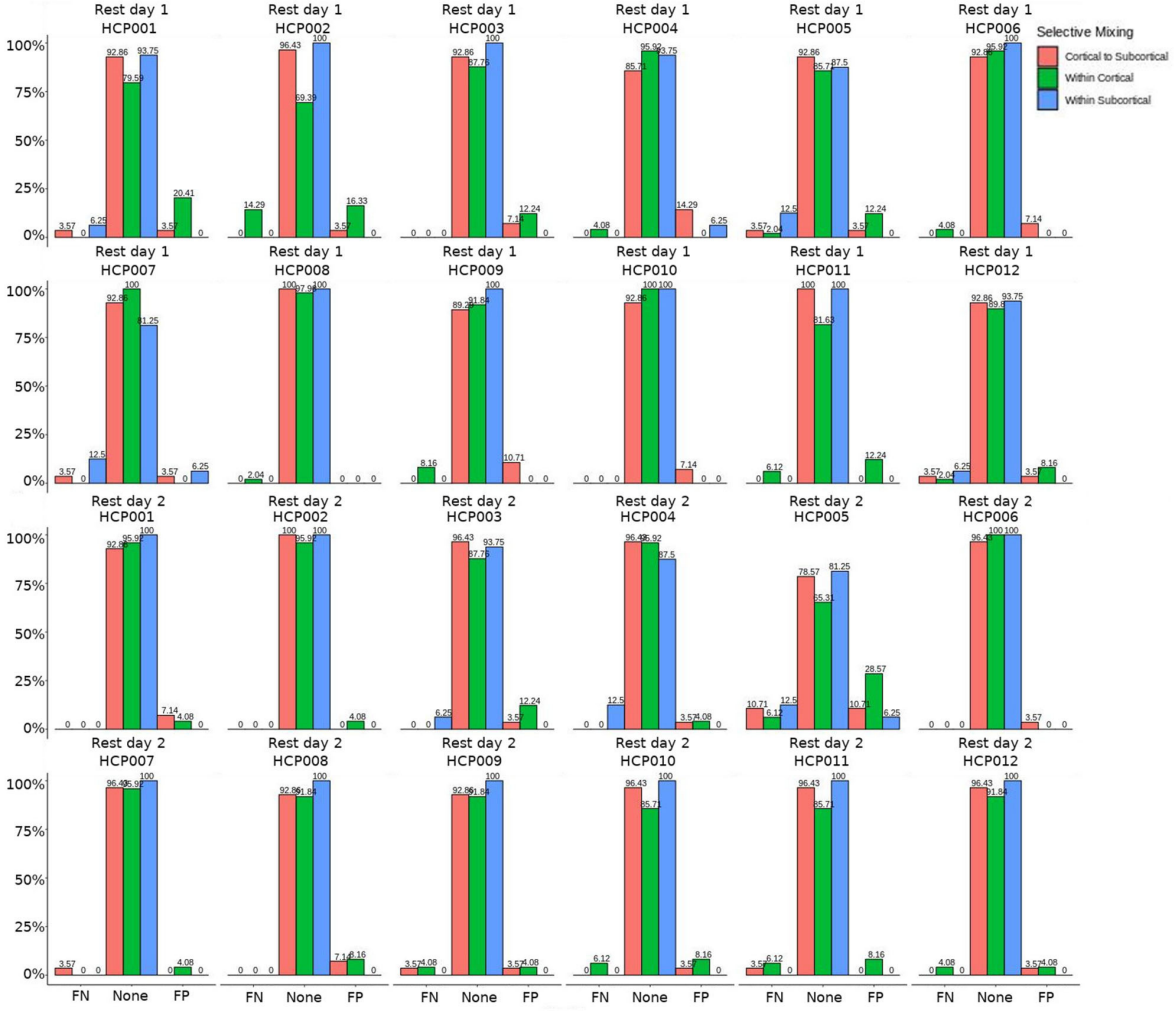
HCP participants comparison for the interaction effect. These are individual participants' FN and FP rates for the selective mixing of the cortical nodal labeling. The FP and FN rates for each participant are quite homogeneous (except for HCP0005), which indicates the group finding of no rate differences are representative of the most participants.

In both HCP participants and patients, we assessed the functional connectivity types with ERGM using four models (i.e., base, structural, GWESP, and complete) to reveal the FPs and FNs that result from the omissions of the structural terms (i.e., the structural effect), triadic closure term (i.e., the GWESP effect), and both structural and triadic closure (i.e., the interaction effect). Leaving out the structural terms, the HCP participants all had either FP or FNs for cortical/subcortical connectivity (see column B in Figure 2. Using multinomial regression we tested for differences in FP and FN rates between their two resting state imaging, the structural effect, GWESP effect and interaction effect comparisons all revealed no significant change in odds ratios for the resting state imaging session performed on day 2 compared to day 1 (see Table 7). Despite these lack of differences in resting state imaging days, all patients had FP or FN rates for leaving out the triadic closure term (see column A in Figure 2, and for the interaction effect of leaving out both the structural and GWESP terms (see Figures 3, 4).

**Table 7:** The effect of daily variability in resting state connectivity on FPs and FNs.

	**Multinomial Regression: Cortical Nodal Labeling**					
**Comparison:**	**Interaction effect**		**GWESP effect**		**Structural effect**	
	**Constant**	**Rest2**	**Constant**	**Rest2**	**Constant**	**Rest2**
False negatives for Cortical to Subcortical	0.00597^***^	1.49			0.00614^***^	1.97
	(0.355)	(0.458)			(0.355)	(0.435)
False positives for Cortical to Subcortical	0.0269^***^	0.716	0.039^***^	0.681	0.0338^***^	0.628
	(0.169)	(0.260)	(0.141)	(0.220)	(0.153)	(0.245)
False negatives for Within Cortical	0.0157^***^	0.614	0.001^***^	0.984	0.937^***^	0.797
	(0.220)	(0.355)	(0.708)	(1.001)	(0.198)	(0.296)
False positives for Within Cortical	0.0299^***^	1.09	0.031^***^	0.937	0.0515^***^	0.869
	(0.160)	(0.222)	(0.157)	(0.224)	(0.125)	(0.183)
False negatives for Within Subcortical	0.00448^***^	0.826	0.002^***^	0.656	0.00307^***^	1.73
	(0.409)	(0.607)	(0.578)	(0.914)	(0.501)	(0.628)
False positives for Within Subcortical	0.00149^***^	0.496	0.002^***^	<0.0001	0.000768^***^	1.97
	(0.708)	(1.23)	(0.578)	(<0.0001)	(1.00)	(1.23)
Observations	2904		2904		2904	
Log Likelihood	-1072.262		-1702.825		-1316.831	
Akaike Inf. Crit.	2168.524		1722.825		2657.662	

* *p < 0.05*;

** *p < 0.01*;

*** *p < 0.001*.

*For the cortical nodal labeling, the FPs and FNs for each type of connectivity pattern were predicted for the first resting state imaging days. The change in odds ratios and their standard errors in parentheses are listed for the interaction, GWESP and structural effect comparisons. Overall, there are no significant change in odds ratios for any of the three model comparisons*.

**Figure 5:**
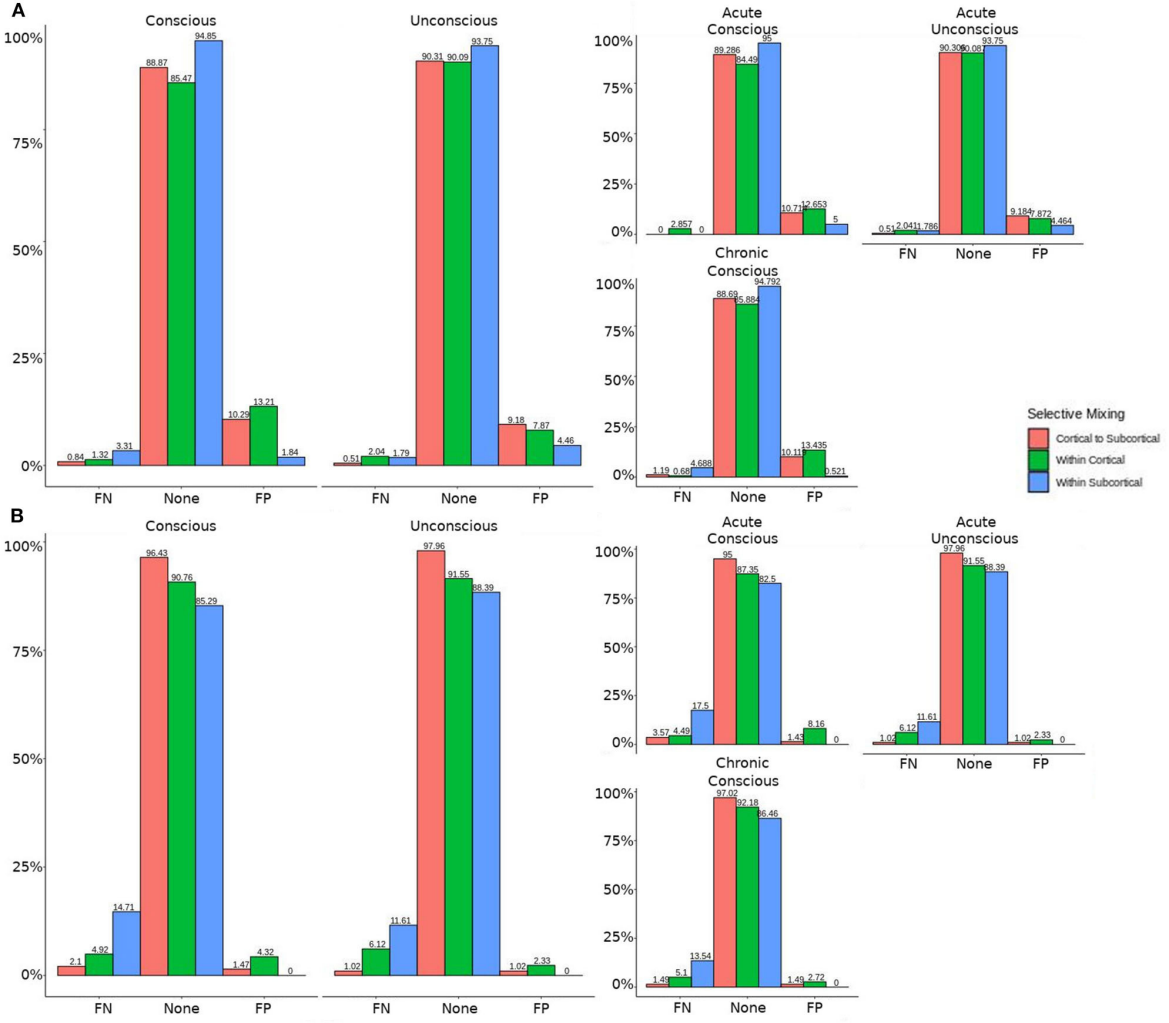
Patients comparison revealing the structural and GWESP effects. For the model comparison of base model (i.e., selective mixing only) to GWESP model (i.e., selective mixing with modeling triadic closure), this figure shows, in row **(A)** the FN and FP rates of resting state network nodes grouped by cortical and subcortical regions when the patients are divided into their level of consciousness assessed by behavioral metrics (i.e., GOS-E). The none category contains both the TP and TN across models. In row **(B)** the effect on the FN and FP rates for leaving out the structural connectivity terms (i.e., structural effect) for the same types of connectivity when the patients are divided into their stage of TBI and their level of consciousness assessed by behavioral metrics. These model comparisons revealed triadic closure effects on the rates of FPs for within cortical connectivity and FNs for within cortical connectivity in unconscious and chronic patients. Additionally, there were structural effects on the rates of FNs for within cortical connectivity and FPs for within subcortical connectivity for chronic patients.

**Figure 6:**
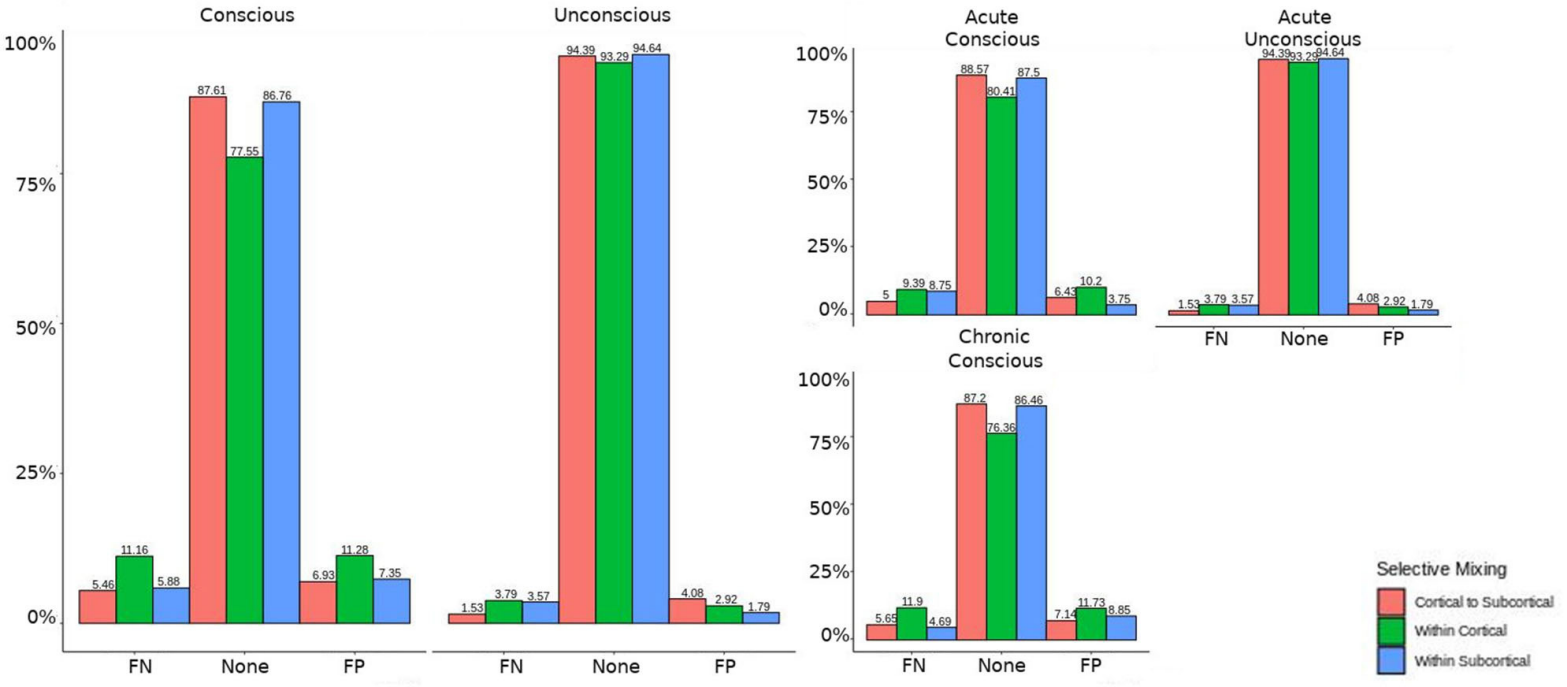
Patients comparison for the interaction effect. When comparing the base model (i.e., selective mixing only) to complete model (i.e., selective mixing with modeling both triadic closure and SC), the FN and FP rates of resting state network nodes grouped by cortical and subcortical regions when the patients are divided into their level of consciousness assessed by behavioral metrics (i.e., GOS-E). The none category contains both the TP and TN across models. The right figure displays the FN and FP for the selective mixing of the cortical nodal labeling when the patients are divided into their stage of TBI and their level of consciousness assessed by behavioral metrics. Overall, This comparison revealed rate differences in cortical or subcortical regions' connectivity patterns between patients with different level of consciousness, but no rate differences in cortical or subcortical regions' connectivity patterns between different stages of TBI.

**Figure 7:**
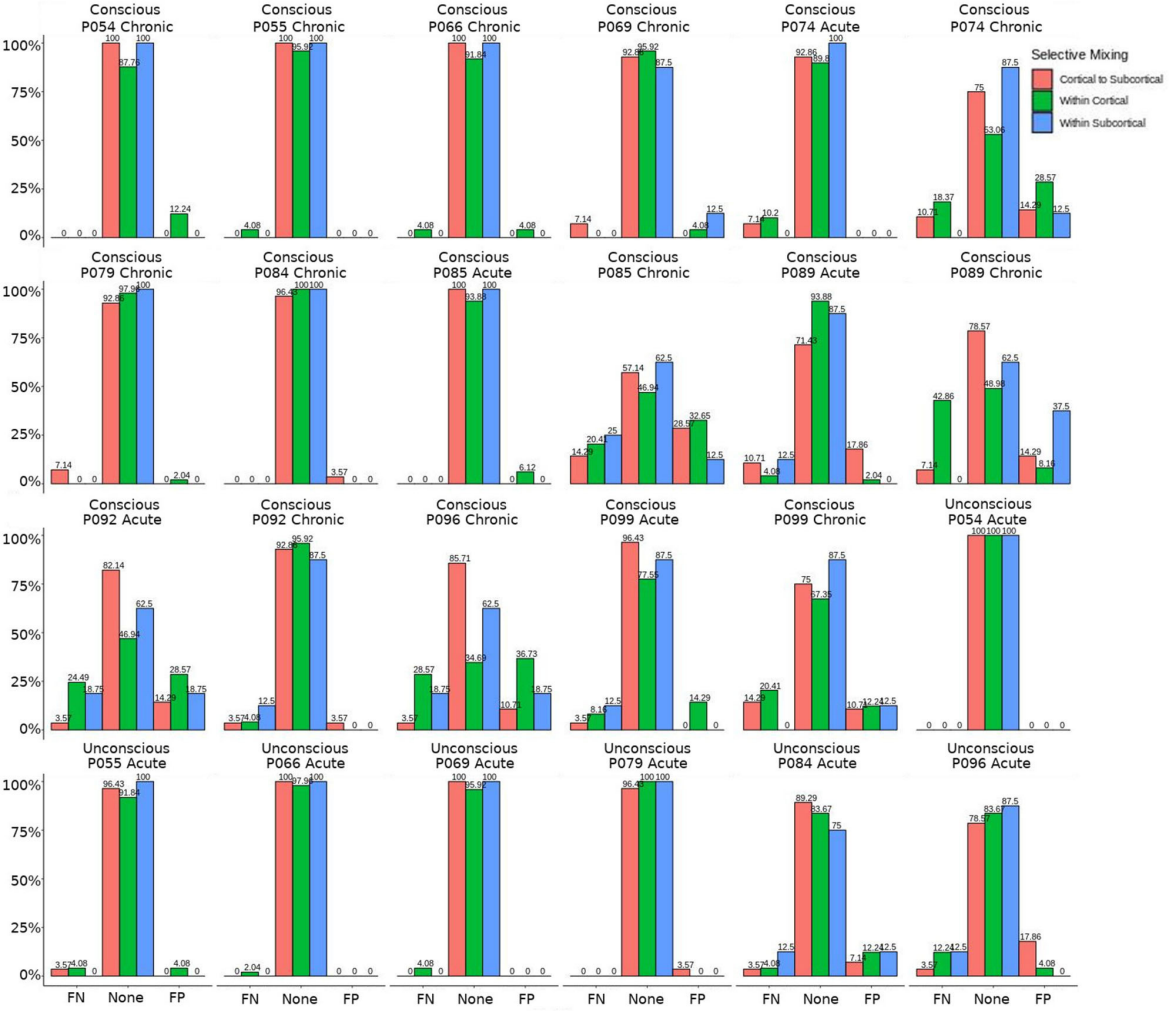
Patients comparison for the interaction effect. These are individual patients' FN and FP rates for the selective mixing of the cortical nodal labeling. The FP and FN rates for each participant are quite heterogeneous. However, most of the conscious patients have large amounts of FNs and FPs, while the unconscious patients have much lower amounts of FNs and FPs, which indicates the group finding of rate differences are representative of the most patients.

The omission of structural terms generate FPs or FNs for all patients (see row B in Figure 5) did produce a 0.401 times decrease in FN for cortico-subcortical connectivity and a 0.321 times decrease for FP in within cortical connectivity for conscious chronic compared to conscious acute stage of TBI patients (see column 3, in Table 8). A similar pattern was found for unconscious patients compared to conscious acute patients, where there was a significant 0.274 times decrease in FN for cortico-subcortical connectivity and a 0.274 times significant decrease for FP in within cortical connectivity. For the GWESP (see row A in Figure 5), only the chronic patients had a significant decrease in FP for within subcortical connecitivty and significant decrease in FP for within cortical connectivity compared to acute patients (see column 2, in Table 8). Finally, the interaction effect of leaving out GWESP and structural terms (see Figures 6, 7) produced FPs and FNs significant decreases in odds ratios of 0.277 times, 0.366 times, 0.259 times, and 0.431 times for FNs in cortico-subcortical connectivity, FNs and FPs for within cortical, and FP for within subcortical, respectively. Overall, the patients with DOC did have differing rates FP and FN and their rates were not driven by a few conscious patients with DOC, rather there were clear high rates of FP and FNs for seven of the seventeen conscious patients (i.e., P074 chronic, P085 chronic, P089 acute, P089 chronic, P092 acute, P096 chronic, and P099 chronic; see Figure 7) compared to none of the seven unconscious patients having rates close to the seven conscious patients.

**Table 8:** The effect of level of consciousness and stage of TBI on FPs and FNs.

	**Multinomial Regression: Cortical Nodal Labeling**								
**Comparison:**	**Interaction effect**			**GWESP effect**			**Structural effect**		
	**Constant**	**Unconscious**	**Chronic**	**Constant**	**Unconscious**	**Chronic**	**Constant**	**Unconscious**	**Chronic**
False negatives for Cortical to Subcortical	0.0272^***^	0.277^***^	1.16	NA	NA	NA	0.0183^***^	0.274^*^	0.401^*^
	(0.271)	(0.491)	(0.317)	(NA)	(NA)	(NA)	(0.319)	(0.594)	(0.450)
False positives for Cortical to Subcortical	0.0350^***^	0.575	1.14	0.05628^***^	0.83284	0.94166	0.00733^***^	0.685	1.00
	(0.240)	(0.348)	(0.281)	(0.188)	(0.254)	(0.225)	(0.502)	(0.709)	(0.594)
False negatives for Within Cortical	0.0447^***^	0.366^***^	1.31	0.01313^***^	0.69403	0.23741^*^	0.0201^***^	1.31	1.09
	(0.213)	(0.352)	(0.246)	(0.380)	(0.537)	(0.629)	(0.305)	(0.376)	(0.356)
False positives for Within Cortical	0.0485^***^	0.259^***^	1.18	0.05816^***^	0.60447	1.05868	0.0366^***^	0.274^**^	0.321^***^
	(0.205)	(0.378)	(0.239)	(0.185)	(0.269)	(0.218)	(0.228)	(0.422)	(0.339)
False negatives for Within Subcortical	0.0136^***^	0.370	0.551	NA	NA	NA	0.0256^***^	0.636	0.746
	(0.381)	(0.629)	(0.507)	(NA)	(NA)	(NA)	(0.271)	(0.389)	(0.335)
False positives for Within Subcortical	0.00583^***^	0.431^*^	2.43	0.00750^***^	0.86763	0.10388^*^			
	(0.579)	(0.915)	(0.628)	(0.502)	(0.673)	(1.12)			
Observations	2904			2904			2904		
Log Likelihood	-1749.64			-1381.539			-1024.384		
Akaike Inf. Crit.	3535.28			2799.077			2078.769		

* *p < 0.05*;

** *p < 0.01*;

*** *p < 0.001*.

*For the cortical nodal labeling, the FPs and FNs for each type of connectivity pattern were predicted for unconscious patients compared to all conscious patients. The change in odds rations and their standard errors in parentheses are listed for the interaction, GWESP and structural effect comparisons. Overall, the interaction effect has a significant decrease in odds ratios for FNs in cortical to subcortical connectivity and within cortical connectivity, and FPs in within cortical and subcortical connectivity*.

## 4. Discussion

Overall, our results highlight two important and overlooked issues in graph analysis. First, brain graphs are susceptible to having different natural levels of density (see Milham et al., 2012; Nielsen et al., 2013) at which they are the most stable and thus, likely, better representations of the structure of the sampled graph. In our data, FC densities ranged from 16.76 to 21.5% for HCP volunteers (see Table 2), and from 10.39 to 15.24% in TBI patients. The use of MoNeT (Narayan et al., 2015), together with ERGM, acknowledges such inter-individual and inter-group differences and views density as a potentially interesting feature of a graph as opposed to a nuisance to be addressed by either imposing an arbitrary level (or range) of density across all graphs (Rubinov and Sporns, 2010; van Wijk et al., 2010) or by assuming fully connected, complex, networks (Rubinov and Sporns, 2011; Fornito et al., 2013, 2016). The issue is all the more important in the context of brain injury and DOC, where full-brain functional and structural connectivity are known to vary across groups (e.g., acute vs. chronic, healthy volunteers vs. patients, coma vs. MCS, etc; Vanhaudenhuyse et al., 2010; Fernández-Espejo et al., 2012; Monti et al., 2015; Zheng et al., 2017; Crone et al., 2018).

Second, our data also show that even when graph density is allowed to vary, failure to account analytically for the interdependence of network measures (i.e., problem #2) and for the structural substrate of functional graphs (i.e., problem #3) significantly affects model estimation. To clarify this issue, it is worth noting that while the conventional approach to graph theory attempts to “summarize” observed networks by measuring their topological properties (e.g., characteristic path length, clustering, and small-worldness Rubinov and Sporns, 2010; Bullmore and Sporns, 2012), ERGM attempts to reconstruct which “social” processes, or combination thereof, are the most likely to have generated the observed networks (hence the term generative processes; Robins et al., 2007; Goodreau et al., 2009). Somewhat analogously to a multiple regression framework (Goodreau et al., 2009), ERGM can thus be viewed as a procedure to determine which combination of generative processes are most likely to explain the observed network (e.g., triadic closure, which is measured by the GWESP term, and captures the degree to which, if node A and node B both connect to node C, A and B more likely to be connected compared to any other two nodes; selective mixing, which is measured by the mixing term, and captures the degree to which nodes that are part of the same group [e.g., hemisphere] more likely to connect with each other). Thus, much like in a conventional multiple regression, omission of informative explanatory variables from the model (i.e., terms) results in incorrect estimation of the association between a dependent variable (which, in ERGM, is the observed network) and each explanatory variable (in ERGM, the outcome metrics associated with the generative processes; for further discussion see Dell'Italia et al., 2018).

The importance of this issue becomes clearer when considering that many of the questions of interest in the context of DOC and TBI are questions about specific generative processes. For example, asking whether cortico-cortical or cortico-subcortical connectivity play different roles in the maintenance or recovery of consciousness (e.g., Laureys et al., 2000a,b; Boly et al., 2009, 2011; Vanhaudenhuyse et al., 2010; Crone et al., 2014, 2018; Amico et al., 2017) is, in the ERGM framework, a question of selective mixing: is level of consciousness associated with greater/smaller proclivity for nodes within cortex to associate preferentially with nodes in the same group (i.e., cortex)? In order to properly answer this question, the model needs to account (i.e., parcel out, keeping the analogy of the multiple regression) for the shared variance of selective mixing and other generative processes (e.g., triadic closure measured by number of triangles in a graph).

Indeed, the present results show that both patients and HCP participants had non-zero rates for FP and FN, if either triadic closure or the structural term were omitted. Crucial for this literature, however, conscious and unconscious patients exhibited very different rates, potentially because, as we discuss more below, of the different effect of mis-modeling on the two populations (due to their different underlying structural/functional properties).

Leaving out a known interrelated measures (e.g., GWESP term for triadic closure term; see column 2 in Table 8) increases risks for differences in rates in FN for within cortical connectivity and FP within subcortical connectivity for comparisons between acute and chronic stage TBI patients. As we discussed in the introduction, triadic closure is one the key generative processes (Goodreau et al., 2009) present most graphs, but more importantly these differences in FP an FN rates could be due to the importance of triadic closure to consciousness. Clustering coefficients have been used to differentiate patients with DOC (using structural connectivity; Tan et al., 2019) and for differentiating levels of consciousness while undergoing anesthesia (Monti et al., 2013). Due to the empirical findings of different levels of triadic closure within patient groups, the best way to avoid these FP and FN rate differences is to model the selective mixing and triad closure processes in a single model.

Similarly, omitting the structural terms (see column 3 in Table 8) affects the FP rates differently for the unconscious patients in their acute stage of TBI and the conscious patients in the chronic stage of their TBI for both within cortical connectivity and cortico-subcortical connectivity. These effects are quite problematic for DOC research due to the interest in comparing cortico-cortico and thalamo-cortical connectivity (e.g., Laureys et al., 2000a,b; Boly et al., 2009, 2011; Vanhaudenhuyse et al., 2010; Crone et al., 2014, 2018; Amico et al., 2017) and its importance to consciousness. Due to the inherent nature of structural damage affecting the structural connectivity, the inclusion of structural terms into a model assessing functional connectivity will help to avoid the FP and FN rate differences between patient in acute and chronic stage of TBI, and the difference in rates between unconscious and conscious patients.

Finally, leaving out both the structural terms and the GWESP term (see column 1 in Table 8) has differing rates in FNs for cortico-subcortical connectivity, FN and FPs for within cortical connectivity, and FPs for within subcortical connectivity. Since all these effects are for unconscious patients, it affects all of DOC research for all three possible types of comparisons between cortical and subcortical connectivity. As can be seen in Table 8, the combination of leaving both terms is not just a simple combination of leaving out each term, but has a specific impact on unconscious patients. A possible explanation is that, for unconscious patients, structural connectivity alterations related to the traumatic injury affects the patients' triadic closure. These differences, however, are might by not accounting for patients' structural connectivity metrics. These types of interactions are key reasons for including all the generative processes in a graph and other key contributing factors such structural connectivity metrics in patient work, particularly when structural pathology is such a prominent phenotype of this cohort (Lutkenhoff et al., 2015, 2019).

### 4.1. Limitations

While we have demonstrated that all three problems would have affected this analysis, we did not fit the data well for the specific effects of triadic closure. The GWESP term matched the overall observed values (see Table 5 for patients, and for HCP participants, see Table 4), but our fits were sub-optimal for most specific edge shared partners (see Table 6). The edge shared partners are a type of triadic closure that measures the number of triangles that share an edge. The effect of these poor fits are not well-understood in our field. There is not any work that we are aware of, which explains specific amounts of triadic closure and their neural mechanisms. There is some work describing larger scale interpretations of outcome metrics (e.g., local and global clustering cofficient; Rubinov and Sporns, 2010, 2011) or to characteristic network blocks (i.e., motifs; Sporns and Kötter, 2004), but these accounts are for general interpretations without linking them to key neural mechanism. More theoretical work is needed to understand how these generative processs (i.e., sociality, selective mixing, and triadic closure) arise from neural mechanisms.

Finally, we only accounted for the structural terms by using measures of centrality and higher order clustering terms (e.g., clustering coefficient and latent space clusters). This is the equivalent of parceling out the effects of structural connectivity on functional connectivity, similar to using covariates in a regression analysis to control for confounding effects. We have not truly estimated the effects of structural connectivity on functional connectivity. There are structural effects which could account for generative processes that we are attributing to the functional connectivity. For example, if a three nodes have two structural edges, does the functional close this triad? A process like this would represent a possible structural and functional relationship from triad closure from the combination of structural and functional connectivity.

## 5. Conclusions and Future Work

For unconscious patients, the lower rate of false positives and/or false negatives for within cortical connectivity, within subcortical connectivity, and cortico-subcortical connectivity reveal the problems of interrelated outcome statistics and leaving out structural connectivity. These effects can result in misinterpretations of selective mixing terms, if the other generative processes are not included. The proper interpretation selective mixing in functional connectivity for unconscious patients are key to understanding how disruptions of functional connectivity can disrupt consciousness. Additionally, the biases of not including all the generative processes will affect any group comparisons between unconscious patients and other patients with a DOC. We suggest future studies in DOC patients model all the generative processes and include structural connectivity metrics when possible.

We did not compare the patients to the HCP data due to the differences in quality of BOLD imaging and imaging parameters. However, we suspect that there would be differences similar to comparing the groups of patients because the underlying cause of these false positives and false negatives is the importance in generating structure in the brain. Patient populations differ in this structure, which would be reflected by difference in sociality, selective mixing, and triadic closure. Leaving out one of these generative processes will affect the rest (Goodreau et al., 2009). Additionally, the structural terms are specifically important to DOC due to the TBI resulting in structural damage that is part of the recovery process. While there may be alternatives to solving the three of the four problems we outlined (i.e., problem#1, #2, and #3), we chose to use exponential random graph models due to the flexibility it provides to model all the generative processes and other covariates of interest (e.g., size of regions of interest or amount of atrophy within an region of interest). This flexible framework would allow researchers to generate models that fit their specific needs and research questions.

In addition, a method is needed that accounts for the social processes that generate the complex interactions between structural and functional connectivity. Structural and functional connectivity are part of a multi-level problem (i.e., there is a structural layer, functional layer within the brain and finally an interaction between these layers). By this, we mean that each have their own generative processes that govern their structure and there are interactions between the levels that drive the brain dynamics. Multi-level exponential random graph models (Wang et al., 2013, 2016; Lazega and Snijders, 2016) have been developing to capture the the nested structure of networks. A concrete example is collaborative research (Lazega et al., 2008), in which, researchers have advice networks for their research problems and the laboratories have collaboration networks. The researchers advice network would be the micro-level network because they are nested within the laboratories (i.e., macro-level). Each of these levels have their own generative processes associated with the structure, but there is a third layer (i.e., the meso-level) that affects both levels. In the collaborative research example, the researchers' affiliation with laboratories are the meso-level. This example could be extended to the structural and functional layers of the brain. The functional layer is the micro-level because it is nested within the macro-level structural layer. The meso-level could be the locations of the functional layer within the structural layer or an estimation of joint functional structural connectivity could be the meso-level (e.g., hybrid connICA; Amico et al., 2017). These multi-level exponential random graph models would allow for a more complete solution to the four problems posed in this thesis because all the levels of functional and structural connectivity could be jointly estimated and properly modeled. This would be a large step in the direction of unraveling the complex interactions between structural and functional connectivity that generate the ability for our complex behavior.

## Data Availability Statement

The datasets generated for this study are available on request to the corresponding author.

## Ethics Statement

The studies involving human participants were reviewed and approved by UCLA Institutional Review Board. The patients/participants provided their written informed consent to participate in this study.

## Author Contributions

JD, MM, and PV designed the experiment. JD and MJ analyzed the data. JD and MM interpreted the results. JD drafted the manuscript. All authors provided the feedback.

## Conflict of Interest

The authors declare that the research was conducted in the absence of any commercial or financial relationships that could be construed as a potential conflict of interest.
